# Spermatogenesis in haploid males of the jewel wasp Nasonia vitripennis

**DOI:** 10.1038/s41598-019-48332-9

**Published:** 2019-08-21

**Authors:** Patrick M. Ferree, John C. Aldrich, Xueyuan A. Jing, Christopher T. Norwood, Mary R. Van Schaick, Manjinder S. Cheema, Juan Ausió, Brent E. Gowen

**Affiliations:** 1 W. M. Keck Science Department, Claremont McKenna, Pitzer, and Scripps Colleges, Claremont, CA 91711 USA; 2 Department of Biochemistry and Microbiology, University of Victoria, Victoria, BC V8W-3P6 Canada; 3 Department of Biology, University of Victoria, Victoria, BC V8W-3P6 Canada

**Keywords:** Developmental biology, Spermatogenesis

## Abstract

Males of hymenopteran insects, which include ants, bees and wasps, develop as haploids from unfertilized eggs. In order to accommodate their lack of homologous chromosome pairs, some hymenopterans such as the honeybee have been shown to produce haploid sperm through an abortive meiosis. We employed microscopic approaches to visualize landmark aspects of spermatogenesis in the jewel wasp *Nasonia vitripennis*, a model for hymenopteran reproduction and development. Our work demonstrates that *N*. *vitripennis*, like other examined hymenopterans, exhibits characteristics indicative of an abortive meiosis, including slight enlargement of spermatocytes preceding meiotic initiation. However, we saw no evidence of cytoplasmic buds containing centrioles that are produced from the first abortive meiotic division, which occurs in the honeybee. In contrast to other previously studied hymenopterans, *N*. *vitripennis* males produce sperm in bundles that vary widely from 16 to over 200, thus reflecting a range of cellular divisions. Our results highlight interesting variations in spermatogenesis among the hymenopteran insects, and together with previous studies, they suggest a pattern of progression from meiosis to a more mitotic state in producing sperm.

## Introduction

Spermatogenesis is one of the most elaborate cell transformational processes in animal development. Much of what is known about spermatogenesis in insects stems from numerous studies in the dipteran fruit fly, *Drosophila melanogaster*. Initially, a single germ cell produced from a stem cell division, termed a gonialblast, undergoes exactly four rounds of mitosis with incomplete cytokinesis, forming a cyst of 16 interconnected germ cells referred to as spermatogonia (also referred to as cystocytes)^1,2^. Following a round of S-phase and enlargement in volume by ~25-fold^1–3^, these cells, now referred to as primary spermatocytes, pass synchronously through the two divisions of meiosis, producing 64 round, haploid spermatids^1,2,4^. The chromatin of these cells becomes remodeled such that the highly basic histone proteins are removed from DNA, and it is repackaged with sperm nuclear basic proteins (SNBPs), including protamines^5^. Each spermatid, including its nucleus, becomes highly elongated as it forms a flagellated tail and a pair of long, tubular mitochondrial derivatives^2^. Grouped in bundles of exactly 64^1,2^, the spermatids undergo individualization through an actin-myosin-dependent process in which they become separated into mature sperm^6–8^.

A central question is to what degree deviations from this general paradigm of spermatogenesis occur in other insects. One outstanding example stems from studies performed in several bee species and a handful of other related insects that pertain to meiosis. In diploids, meiosis involves the side-by-side alignment of homologous chromatid pairs and the physical exchange of genetic material between them during the first meiotic division^9^. Interestingly, in hymenopteran insects, which includes all bees, ants, wasps and sawflies, males arise as haploids from unfertilized eggs (reviewed in^10^). To be clear, males of these organisms contain only a single set of chromosomes inherited from the maternal parent. This characteristic makes homologous chromatid alignment and recombination in the male germ line impossible, thus begging the question of how haploid males produce sperm.

Previous work has shown that in the honey bee, *Apis mellifera*, like in *D*. *melanogaster*, the primary spermatocytes undergo cellular enlargement before entry into meiosis^11^. However, interestingly, in *A*. *mellifera* the nuclear envelope fails to break down in each spermatocyte during meiosis I even though the chromatin condenses into chromatids^11,12^. Each spermatocyte becomes clavate, or tear-dropped, in shape as it appears to undergo division. However, this division fails to occur, resulting in the original primary spermatocyte as the product. In the Indian honey bee *Apis cerana indica*, the first meiotic division does indeed occur, but the products consist of a large daughter cell containing the undivided nuclear material and a much smaller “cytoplasmic bud” containing no nuclear material^12^. Additionally, previous TEM studies in *A*. *mellifera* and other bees have noted the presence of small cytoplasmic buds containing extra, unused centrioles produced before or during the abortive division^11–13^. The second meiotic division in these insects is more conventional, being symmetrical and involving complete nuclear envelope breakdown and sister chromatid separation, resulting in haploid spermatids that subsequently undergo the final stages of spermatogenesis. It is presumed that the abortive division during meiosis I is an alteration of the basic cellular meiotic programming that compensates for the lack of homologous chromosomes in haploid males that is needed to properly produce haploid sperm – *i*.*e*., the reductive division step is eliminated because it is neither necessary nor desirable in haploid individuals.

Although abortive meiosis I may be a common feature of spermatogenesis in hymenopteran insects, there appear to be some notable variations in aspects of spermatogenesis that have been found in a few examined species. For example, early TEM studies in males of the jewel wasp, *Nasonia vitripennis*, noted a less pronounced cellular increase of primary spermatocytes than occurs in *A*. *mellifera*^14^. Additionally, the number of sperm per bundle, and, therefore, the number of germ cell divisions producing sperm, varies among different species. For example, a predatory wasp *Microstigmus arlei* has 32 spermatids per bundle (5 divisions)^15^, some bee species have 32 spermatids per bundle while others have 64 spermatids per bundle (5 or 6 divisions)^13^, and the rose sawfly *Arge pagana* has either 256 or 512 spermatids per bundle (7 or 8 divisions)^16^. These patterns seem to follow a 2^n^ rule where n is the number of germ cell divisions giving rise to sperm^17^. An interesting question is what cellular factor(s) determine the number of germ cell divisions for a given species.

These noted cellular variations in spermatogenesis, and the fact that this developmental process has been limited primarily to TEM studies in only a few hymenopteran species, led us to further investigate this process in the jewel wasp *N*. *vitripennis* using a combination of several different microscopic approaches. *N*. *vitripennis*, a parasitoid of different blowfly species^18,19^, has gained prominence over the past two decades as a rising model insect for genetic and genomic studies aimed at understanding the evolution and development of axis patterning^20,21^, sex determination^10,22^, *de novo* centrosome formation^23^, host-symbiont interactions^24–26^, and venom production^27,28^. Additionally, recent work has focused on a paternally transmitted B chromosome known as PSR (for Paternal Sex Ratio), which, through an unknown mechanism, causes complete elimination of the paternal (sperm-derived) half of the *N*. *vitripennis* genome during the first embryonic mitotic division^29,30^. Investigating these biological processes in *N*. *vitripennis*, particularly those involving the male germ line, would be greatly facilitated by a better understanding of spermatogenesis in this organism.

Our study here reveals certain cellular characteristics supporting the likelihood that *N*. *vitripennis*, like other previously studied hymenopterans, undergoes an abortive first meiotic division to produce sperm. However, we observed several unique characteristics of spermatogenesis in *N*. *vitripennis*, including a relatively small increase in primary spermatocyte size compared to *A*. *mellifera*, and a highly variable number of spermatids per bundle. We propose that these and other characteristics may reflect an evolutionary progression from meiosis toward a fully mitotic state of sperm production in *N*. *vitripennis*.

## Results

**Figure 1:**
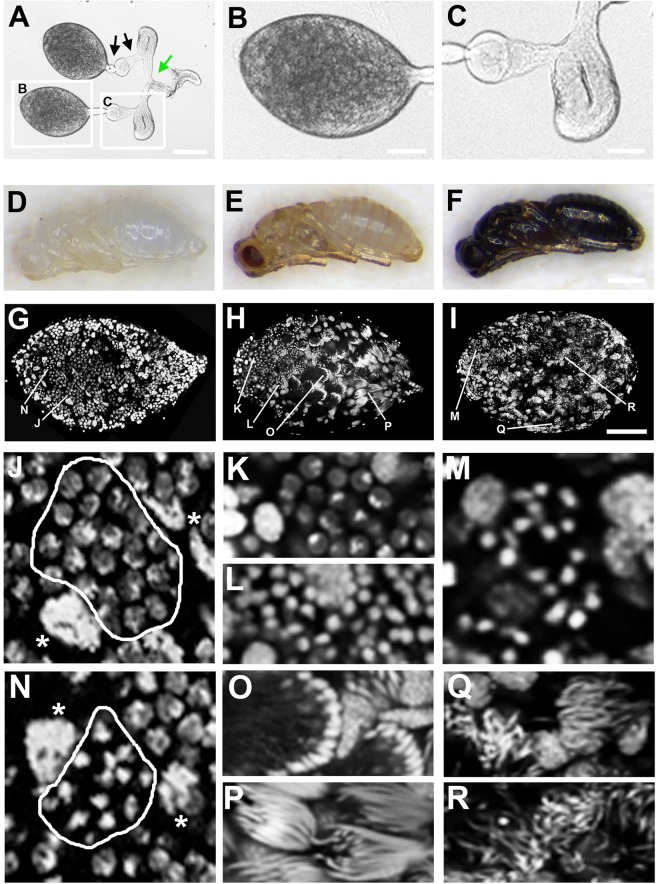
Male reproductive tract and cells within the testis of *N*. *vitripennis*. The bilaterally symmetrical male reproductive tract is shown in (**A**), with the testis (**B**) and seminal vesicle and accessory gland (**C**) shown at higher magnification. In (**A**), the black arrows indicate ducts joining the testis with the seminal vesicle, and the seminal vesicle with the accessory gland, while the green arrow depicts the ejaculatory duct. (**D**–**F**) Three major pupal stages are shown: (**D**) white body stage; (**E**) yellow body/red eye stage; (**F**) fully pigmented black body stage. (**G**–**I**) Fixed testes from these three pupal stages shown at low magnification. Panels (J–R) are high magnification images taken from the three testis shown in (**G**–**I**), with all images pertaining to each testis shown below each testis’ column. (**J**) Spermatogonia at interphase. Circumscribed cystocytes are in interphase. (**N**) Spermatogonia with condensed chromosomes. The chromatin of circumscribed cystocytes appear condensed into chromosomes. Asterisks in (**J**,**N**) denote trophocyte nuclei. (**K**) Small cyst of spermatogonia at interphase. (**L**) Large cyst of germ cells with condensed nuclei, indicating their entry into spermiogenesis. (**O**) A cyst forming a ‘cup-like’ structure at the onset of spermiogenesis. (**P**) Bundles of elongating spermatid nuclei. (**M**) Germ cells beginning spermiogenesis but not yet in the ‘cup’ formation. (**Q**,**R**) fully elongated sperm nuclei. DNA is grey in panels (G–R). White arrows indicate trophocytes. Scale bar equals 150 μm in (**A**), 60 μm in (**B**), 75 μm in (**C**), 500 μm in (**F**), and 80 μm in (**I**).

**Figure 2:**
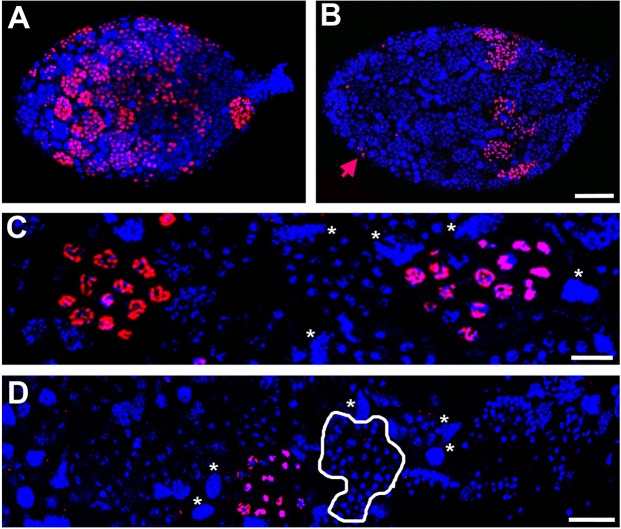
Spermatogonial division is asynchronous across different cysts. (**A**) A testis from a white/yellow body pupa containing numerous cysts with germ cells undergoing division, as indicated by the presence of H3S10p (red). (**B**) A testis from a yellow body-red eye pupa containing a narrow region of cysts with germ cells undergoing division. The germ cells of most cysts in this testis are not undergoing division. However, a few single cells at the testis anterior appear to be dividing (red arrow). (**C**) A high magnification of a region within a testis containing two cysts undergoing division. Resolved chromosomes can be seen with high levels of H3S10p. (**D**) A high magnification of a region within a testis containing one cyst with germ cells undergoing division, in addition to several cysts with round, condensed nuclei and no H3S10p, indicating entry into spermiogenesis. Circumscribed cystocytes have condensed nuclei but are not H3S10p-positive, indicating that they have entered into spermiogenesis. Asterisks in (**C**,**D**) indicate some of the trophocyte nuclei. DNA is blue in all panels. Scale bar equals 40 μm in (**B**), 6 μm in (**C**), and 10 μm in (**D**).

To investigate spermatogenesis in *N*. *vitripennis*, we examined the male reproductive tract during wasp pupal development, when much of spermatogenesis occurs^31^. The male reproductive tract of *N*. *vitripennis* is bilaterally symmetrical, with each of the two sides beginning with an oval-shaped testis that connects at its base through a duct to a seminal vesicle, and then to an accessory gland, before joining to the ejaculatory duct (Fig. 1A–C; see also^32^). The two sides of the male reproductive tract connect at a position that is distal (*i*.*e*., further away from the testis) to the two accessory glands (Fig. 1A). Our analyses henceforth focused on cells within the testis. We previously reported the presence of two major cell types in the wasp testis: (*i*) germ cells organized into cysts, and (*ii*) individual cells that contain very large nuclei and are interspersed among the cysts of germ cells (Figs 1, 2)^33^. These latter cells, known as trophocytes, have been reported previously in bees^34^. We first characterize the cellular behavior of the germ cells before describing the trophocytes.

### Semi-coordinated development of sperm across the nasonia testis

It is known that *N*. *vitripennis* males produce all of their sperm before reaching adulthood^31^. An important question is to what degree sperm formation in *N*. *vitripennis* is synchronized within the testis. At the beginning of pupal development, the testis contains cysts composed of 16 or more germ cells, demonstrating that several divisions have occurred by this time (Fig. 1G,J,N). Unfortunately, we were unable to obtain testes from the larval stage preceding pupal development, likely due to their very small size and difficulty in identifying them within the large amounts of fatty tissue present in late larvae. However, we can conclude that the gonialblasts produce cystocytes that undergo several rounds of division before the pupal stages.

In slightly older testes, the germ cells were more abundant within each cyst, indicating that these cells continue to undergo subsequent divisions (Fig. 1L). Additionally, the nuclear morphology of the germ cells appeared distinct in cysts that are positioned in different locations across the testis. For example, in most cysts located within the apical two thirds of the testis, the germ cells are in interphase (Fig. 1J). However, in some cysts, the chromatin of their cells appears to be resolved into chromosomes, suggesting that these cells are undergoing division (Fig. 1N). To confirm this possibility, we stained testes with an antibody that recognizes histone H3 phosphorylated at Serine residue 10 (H3S10p); this mark appears on condensed/resolved chromosomes and is, therefore, a good indicator of dividing cells^35,36^. Indeed, in these particular cysts the nuclei were H3S10p-positive, whereas the germ cells with uncondensed chromatin in other cysts showed no nuclear H3S10p staining (Fig. 2). Within each H3S10p-positive cyst, all nuclei were H3S10p-positive – that is to say, all of these germ cells appeared to be dividing – thus demonstrating cell cycle synchrony within each cyst (Fig. 2A–D). However, the fact that cells in only a subset of cysts were undergoing division in a given testis (Fig. 2A–D) demonstrates that there is no cell cycle synchrony among the cysts. In only a few cases did we observe individual, H3S10p-positive cells at the apical border (Fig. 2A,B), suggesting that there may be persisting stem cells at this stage. However, these cells are likely to be minimally mitotically active by this time.

**Figure 3:**
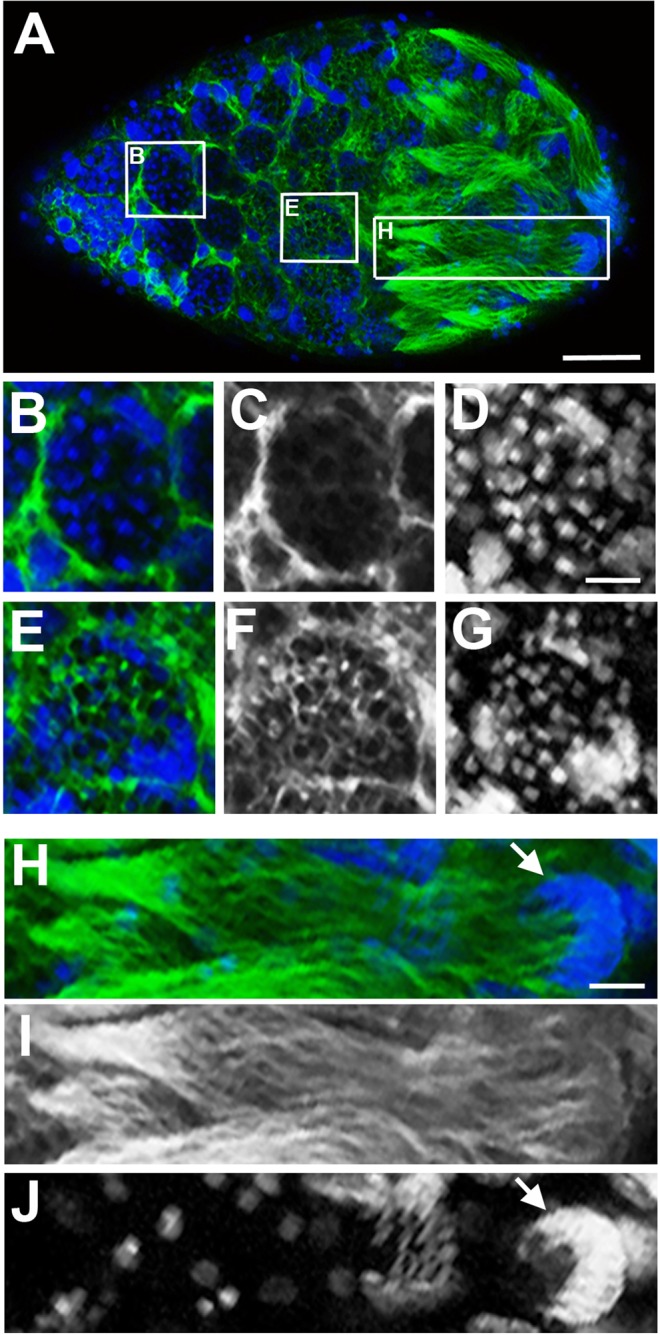
Sperm tail formation in the *N*. *vitripennis* testis. Regions in the testis of yellow body-red eye pupa devoid of nuclei (**A**) are filled with extending sperm tails (microtubules are green; DNA is blue). (**B**–**D**) Microtubules are enriched outside but not inside of cysts at the apical end of the testis. (**B**) is a merge; (**C**) is microtubules in greyscale; (**D**) DNA in greyscale. (**E**–**G**) Germ cells in a more posterior-located cyst have entered into spermiogenesis, as indicated by their condensed nuclei. The microtubules have begun to become enriched within each cell (**E** is a merge; **F** is microtubules in greyscale; **G** is DNA in greyscale). (**H**–**J**) A bundle of spermatids with elongating tails, whose ends coalesce into a ‘cone’ like structure. (**H**) is a merge of microtubules in green and DNA in blue. The arrow in (**H**,**J**) indicates the elongating nuclei that are tightly organized into a cup. (**I**) Microtubules is in greyscale; (**J**) DNA is in greyscale. Scale bar equals 50 μm in (**A**), 10 μm in (**D**), and 15 μm in (**H**).

In cysts located near the distal end of testes from older pupae (*i*.*e*., in the late yellow-red and the yellow-black stages), the nuclei appeared small, round, and hyper-condensed (Figs 1L,M; 2C,D) but they were not H3S10p-positive (Fig. 2C,D). In a subset of these cysts, the cells became reorganized into a hemispherical shape, with the “cup” oriented toward the testis’ distal end (Fig. 1H,O). In addition, we observed regions of negative space between the cysts (Fig. S1). Staining with antibodies that recognize acetylated alpha-Tubulin revealed that these spaces are filled by newly forming tails (Fig. 3A), a process that occurs during spermiogenesis. Additionally, the nuclei within these cysts become elongated at this time (Figs 1P; 3H–J). For these reasons, we refer to these germ cells as spermatids. Broadly, the cysts appear to develop in a manner that is not strictly synchronized with one another with regard to cell division or stage of spermatogenesis, although the cysts located at differing ends of the testis do not differ greatly regarding the specific stage of spermatogenesis (Fig. 1G,H). Thus, the cysts are semi-synchronous with one another across the testis, although each cyst appears to have its own cell cycle. In testes of the most advanced (black) pupal stage, the germ cells in the majority of cysts have either entered into spermiogenesis, as indicated by their condensed and/or elongating nuclei (Fig. 1M), or they have elongated into spermatid bundles (Fig. 1Q,R).

### Evidence of ‘abortive’ meiosis in *N. vitripennis*

We examined *N*. *vitripennis* testes for cellular characteristics indicative of abortive meiosis I, which typifies spermatogenesis in bees^12,13^. For this purpose, we focused on testes from pupae in the yellow-red stage; testes at this stage contain dividing germ cells at the anterior and those that have entered spermiogenesis, and therefore are most likely to include meiotic divisions. Despite seeing multiple H3S10p-positive cystocytes during these stages through confocal analysis (Fig. 2), we saw no cystocytes with condensed chromatin in our TEM images. Therefore, we were unable to observe direct evidence of an abortive meiosis I division. Additionally, we saw no evidence of cytoplasmic buds, nor did we see germ cells were clavate-shaped, as has been reported for bee species^11–13^.

**Figure 4:**
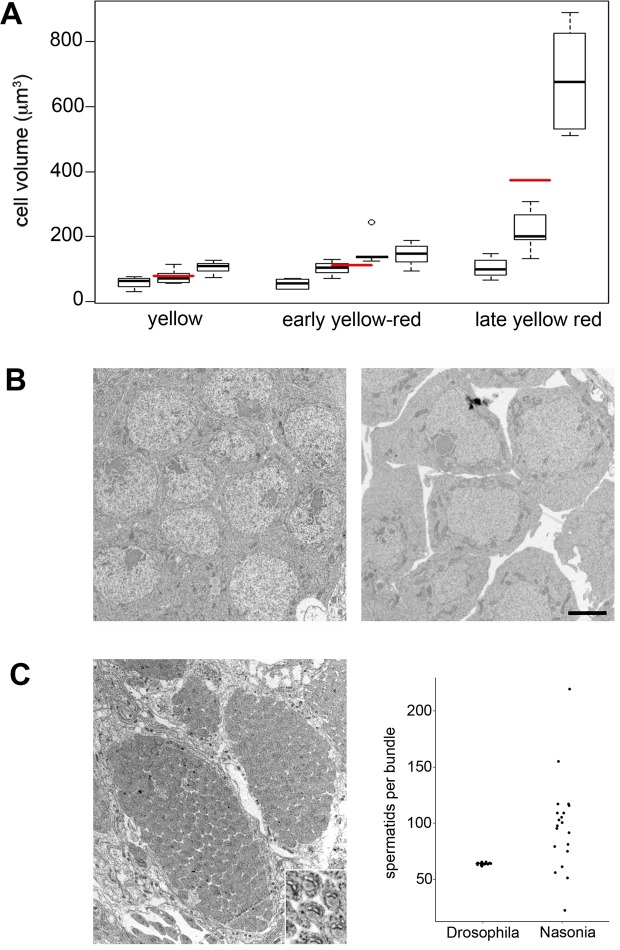
Wasp spermatogenesis produces bundles with highly variable spermatid numbers. (**A**) Cystocyte volume averages across three different pupal stages. Dark black lines represent means for a given cyst. Red lines represent means for all cyst means within each pupal stage. Boxes depict the interquartile range for each each cyst mean, and the whiskers are 1.5 times the interquartile range. (**B**) Left, a cyst of germ cells taken from a testis of an early yellow-red male pupa. Right, a cyst of germ cells taken from a testis of a late yellow-red male pupa. Scale bar is 2 μm and applies to both panels. (**C**) Left, a cross section taken from the posterior end of a testis from a yellow-black male pupa depicting three spermatid bundles, each containing a different number of spermatids. Inset is a high magnification of the sperm tails showing cross sections of the mitochondrial derivatives and axonemes for a cluster of spermatids. Right, a graph showing the number of spermatids per bundle scored for *D*. *melanogaster* testes and *N*. *vitripennis* testes. Scale bar in (**B**) is 2 μm (also applies to (**A**)).

However, several lines of secondary evidence support the notion that *N*. *vitripennis* germ cells do exhibit meiosis-like behavior. First, germ cells preceding entry into spermiogenesis undergo an increase in volume of ~10-fold, based on comparison of the smallest germ cell average observed in yellow pupal testes to the largest germ cell average seen in late yellow-black pupal testes (Fig. 4A,B). Notably, this increase is smaller than values measured for *A*. *mellifera* (~16-fold)^11^ and *D*. *melanogaster* (25-fold)^1,2^. Second, taking advantage of previously published transcriptome data for *N*. *vitripennis* germ line tissues^37^, we looked to see if any genes whose functions are restricted to meiosis are expressed in the wasp testis^38^. Five of eight such genes – DMC1, HOP2, MND1, MSH4, and SPO11 – are expressed at comparable levels in both the testis and ovary (Table S1). One gene, REC8, is expressed over ten-fold higher in the testis than in the ovary (Table S1). A seventh gene, MSH5 is not expressed in either tissue, and the eighth gene, CORT, is expressed at only trace amounts in the testis and ~50 to 100-fold-higher levels in the ovary (Table S1). Thus, almost all of the known meiosis-specific genes are expressed in the wasp testis.

### An unusually variable number of sperm per bundle

In order to estimate the number of cell divisions that occur before spermiogenesis in *N*. *vitripennis*, we counted the number of spermatids contained within individual bundles depicted in TEM images. We carefully scored cell number per bundle by counting cross sections through tails or through nuclei, making sure that each section contained the entire bundle. Interestingly, we observed a wide range in cell number, varying from 15 to 225, per bundle (Fig. 4C). Because the analyzed cross sections were obtained from bundles of spermatids, there would have been no further cell divisions by that time, thereby assuring that our counts reflect final cell numbers per bundle. Thus, we conclude that the number of divisions that produces sperm in *N*. *vitripennis* is highly variable, ranging from at least 4 to 8, a striking departure from the much narrower range of divisions seen in other hymenopteran insects. The numbers of spermatids per bundle did not exactly match multiples of two, as is expected if the germ cell divisions follow a power of 2 division pattern. This deviation in spermatid number may be due to random cell death during the divisions.

### Nuclear changes during spermiogenesis

**Figure 5:**
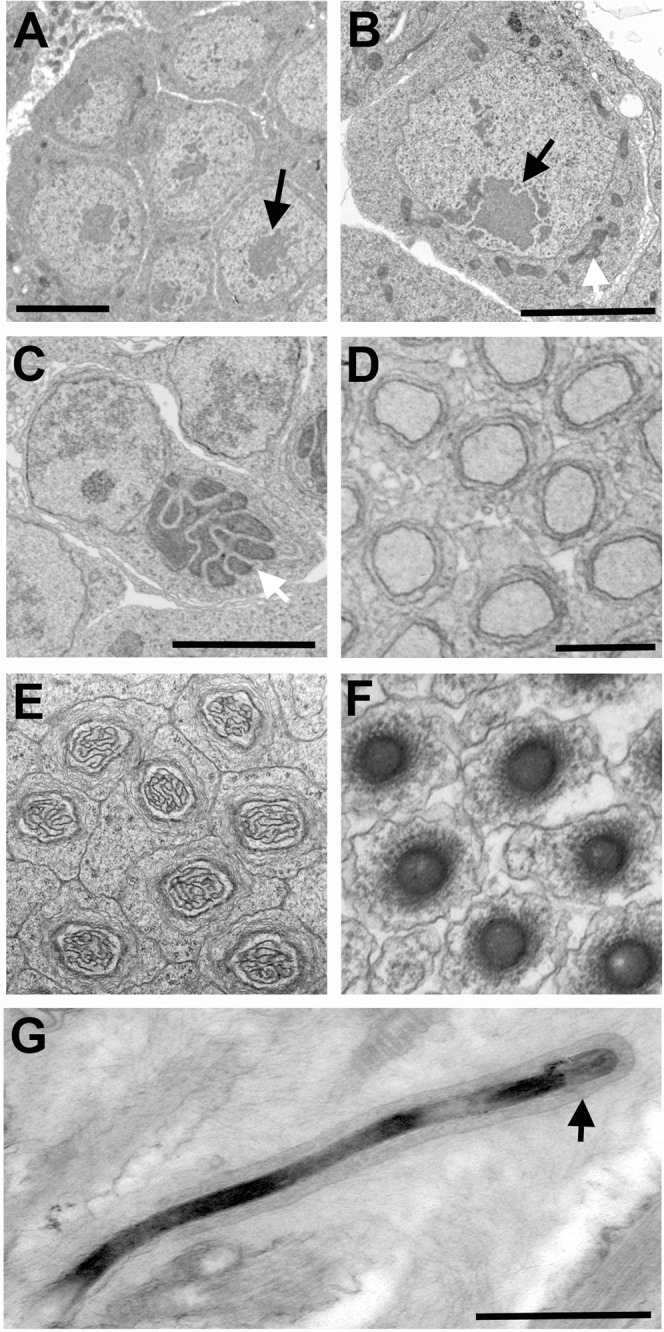
Nuclear changes of germ cells during spermatogenesis. (**A**) A region of a cyst of germ cells (cystocytes) in the testis of a yellow male pupa. (**B**) A germ cell in the testis of a yellow-red male pupa, showing mitochondrial bodies accumulating around the nucleus (white arrow). (**C**) A germ cell with mitochondria that have coalesced into an ‘onion’ like structure called nebenkern (black arrow). Dark grey area within the nucleus of each cell in (**A**–**C**) is the nucleolus (indicated by black arrows in (**A**,**B**)). (**D**) Cross section of nuclei within a bundle of germ cells that have entered into spermiogenesis. The chromatin adjacent to the plasma membrane is becoming electron dense (black) (**E**) Cross section of nuclei within a bundle of spermatids whose chromatin contains thick fibrillar structures. (**F**) Cross section of late spermatids, whose chromatin is very electron dense; by this time, the diameter of the nuclei has decreased substantially. (**G**) An individual sperm with electron dense chromatin, and an acrosome at its tip (black arrow). Scale bars are 2 μm in (**A**), 3 μm in (**B**), 2 μm in (**C**), 1 μm in (**D**), and 500 nm in (**G**) The scale bar in (**D**) also applies to (**E**,**F**).

**Figure 6:**
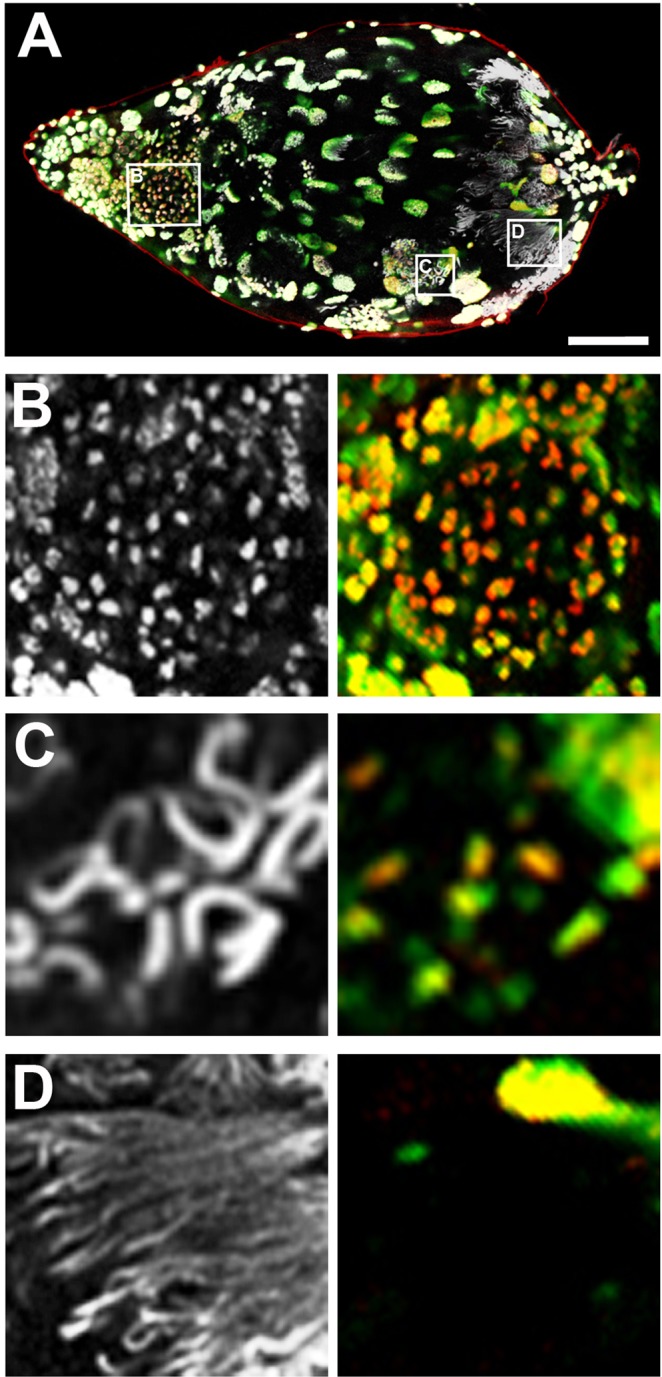
Histone removal during spermiogenesis. In all panels, DNA is grey, green is pan histones, and red is H3K9me3. (**A**) Testis from a yellow-black male pupa. (**B**) A cyst of dividing germ cells located toward the testis’ anterior end. The chromatin of these cystocytes contains histones and H3K9me3. (**C**) A group of individualizing spermatids showing reduced levels of histones and H3K9me3 in small regions in each spermatid. (**D**) Individualized sperm, showing no traces of histones and H3K9me3. Scale bar in (**A**) is 75 μm.

We scrutinized TEM preparations of the testis in order to visualize the nuclear dynamics that occur before and during spermiogenesis. In early cysts, the germ cell nuclei contained chromatin that is of uniform density, with the exception of regions of heightened electron density that are nucleoli (Fig. 5A). During this time, mitochondria are sparsely present in the cytoplasm. In germ cells of slightly older cysts, the mitochondria appear more abundant around the nucleus (Fig. 5B). Subsequently, the mitochondria coalesce into the onion-like agglomerate known as nebenkern (Fig. 5C). During subsequent progression through spermiogenesis, the nuclei of the early spermatids underwent dramatic reshaping. Specifically, these nuclei become elongated, thus shrinking to a diameter of 80–100 nm (Fig. 5D–F). During nuclear elongation, the spermatid’s chromatin begins to appear electron dense around the nuclear periphery (Fig. 5D). In slightly older cells, these dense areas increase and become organized into thick fibrillar structures, reflecting reorganization of the chromatin (Fig. 5E). Once the nuclei of spermatids become fully elongated, their chromatin appears almost uniformly electron dense (Fig. 5F,G), probably reflecting the removal of its histones and repackaging of DNA with SNBPs. We confirmed this idea by visualizing histones in whole testes with fluorescence confocal microscopy. Histones were clearly detectable in cystocytes (Fig. 6A,B) but disappeared altogether in older spermatids and mature sperm (Fig. 6A,C,D). A similar pattern was observed for the histone mark, trimethylation of histone H3 at Lysine residue 9 (H3K9me3) (Fig. 6A–D). These patterns suggest that, similar to other organisms, the sperm’s DNA in *N*. *vitripennis* is stripped of its histones and repackaged with SNPBs during spermiogenesis.

### Nuclear characteristics of trophocytes

**Figure 7:**
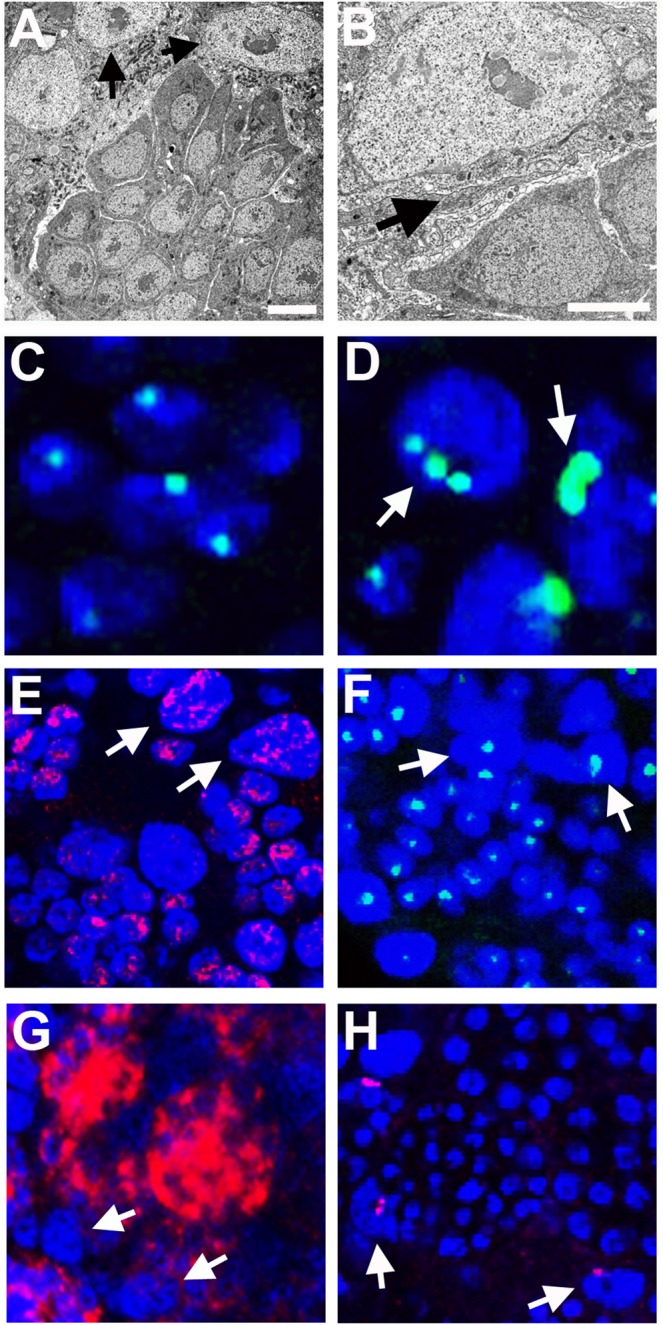
Nuclear characteristics of trophocytes. (**A**) Trophocytes (black arrows) adjacent to cystocytes in the testis of a yellow male pupa. (**B**) High magnification of a trophocyte (top of panel) located adjacent to cystocytes (bottom). Some physical space separates the plasma membranes of these cell types (black arrow). (**C**) A cyst of germ cells stained for rDNA (green); each cystocyte has a single focus of rDNA. (**D**) Several trophocytes, each with multiple foci of rDNA, indicating polyploidy. (**E**) Both trophocytes and cystocytes stained for active RNA polymerase II (red). (**F**) Ribosomal RNA (green) is expressed in both cystocytes and trophocytes. (**G**) Transcripts of the germ-line determinant gene, vasa, are expressed only in cystocytes (red). (**H**) Non-coding transcripts expressed by the B chromosome, PSR, are expressed in trophocytes and not in cystocytes. White arrows in panels (D) through (**H**) point to trophocytes.

We further examined trophocytes to better understand their characteristics. These cells are scattered uniformly throughout the testis and do not appear to change in abundance from early to later pupal stages (Figs 1,2). We found no evidence that trophocytes physically connect with the germ cell cysts, although they are often adjacent to and sandwiched between multiple different cysts (Fig. 7A,B). As a result, trophocytes can be distorted into irregular shapes. The diameter of trophocyte nuclei is quite large, relative to those of the germ cells (Fig. 7B). Indeed, the nuclei of trophocytes take up nearly their entire cellular volumes (Fig. 7B). Previous work employing DNA fluorescence hybridization (FISH) suggested that trophocytes in *N*. *vitripennis* are polyploidy^33^, a feature that is consistent with their large nuclear size. We confirmed these earlier observations by DNA FISH with a probe that marks the single rDNA locus; as with probes to other loci^33^, multiple distinct loci of rDNA could be seen in these cells, whereas a single locus of rDNA was present in each germ cell (Fig. 7C,D). The polyploid nature of trophocytes would be expected to preclude any division of these cells after their entry into endoreplication. Consistent with this idea, trophocytes never exhibited the H3S10p mark, unlike the dividing germ cells (Fig. 2).

The fact that trophocyte nuclei are polyploid suggests that these cells may produce RNAs and proteins important for progression of germ cells through spermatogenesis. Indeed, both trophocyte nuclei, and germ cell nuclei before entry into spermiogenesis, cytologically showed distinct regions of active RNA Polymerase II (Fig. 7E; ref). To further explore the transcriptional behavior of the trophocytes, we used RNA FISH to test for expression of a few genes. Ribosomal RNA was expressed in both trophocytes and germ cells (Fig. 7F). In contrast, transcripts expressed from vasa, a gene that encodes a determinant of germ cells, was expressed only in the germ cells (Fig. 7G). Finally, a transcript corresponding to a non-coding sequence carried by the B chromosome PSR was expressed only by trophocytes (Fig. 7H). Taken together, these characteristics demonstrate that trophocytes are indeed transcriptionally active but they have an expression profile that is somewhat distinct from the germ cells.

## Discussion

In this study we have performed microscopic analyses of developing sperm in the jewel wasp *N*. *vitripennis*, with particular emphasis on the morphology of nuclear changes. Like all other hymenopteran insects, *N*. *vitripennis* reproduces through haplo-diploidy, in which males are haploids – *i*.*e*., they only have a single chromosome set that is derived from the maternal parent. Numerous, interesting cellular differences exist among insects with regard to spermatogenesis; indeed, a distinguishing feature of hymenopteran spermatogenesis is the presence of an abortive first meiotic division. Abortive meiosis can be considered as a requirement for haploid cells that are programmed to follow a meiotic pattern but that only have one set of chromosomes and therefore cannot undergo two meiotic divisions and still produce haploid gametes.

It is likely that *N*. *vitripennis* is no exception to this hymenopteran characteristic of abortive meiosis. First, *N*. *vitripennis* males do indeed produce haploid gametes. Additionally, these males express at least six conserved genes known to function exclusively in meiosis in the testis, and we observed cellular enlargement that is typical of spermatocytes before entry into the first meiotic division^1,2,11^. However, one meiosis-specific gene, CORT, is only expressed in trace amounts in the testis, and at substantially higher levels in the ovary. It is likely that all of these meiosis genes are needed in the female germ line because female wasps are diploid and production of their gametes involves homologous chromosome pairing, recombination, and true meiotic divisions^31^. Reduced or near-absent expression of CORT in the wasp testis may result from a lack of selective pressure to maintain expression of this gene in the male germ line. Moreover, its lack of expression may be directly advantageous by preventing the first meiotic division from occurring, thus insuring proper haploid gamete formation.

Moreover, *N*. *vitripennis* differs from other hymenopterans studied so far in several noteworthy ways regarding spermatogenesis. First, despite that cellular enlargement of germ cells occurs, the degree of enlargement is slightly less than that observed for spermatocytes of *A*. *mellifera*^11^ and much less than for those of *D*. *melanogaster*^1,2^. Second, the number of spermatids per bundle spans a much wider range than occurs in other studied hymenopterans^13,15,16^. An early EM study reported the presence of 128 spermatids per bundle in *N*. *vitripennis*^14^. Unfortunately, this study did not portray clear images of sperm bundles that are assessable to the readership^14^. In our current study, we scored spermatid bundles that contained ~128 spermatids, as well as those containing a much wider range of cell numbers (Fig. 4). It is possible that variation of spermatids per bundle was missed by the earlier study. In any case, these observations suggest a relaxation of regulation for a set number of mitotic divisions producing sperm in *N*. *vitripennis*. And third, we saw no cytoplasmic buds, which are normally produced by the abortive meiotic division. Together, these observations suggest an interesting possibility – that sperm production in some hymenopterans like *N*. *vitripennis* may be transitioning from meiosis to an entirely mitotic state. Such an idea, although speculative, is consistent with the fact that production of sperm starting from a haploid state (*i*.*e*., a single set of chromosomes) would produce haploid gametes, regardless of how many mitotic divisions occur in any given cyst.

Our observations show that the trophocytes interspersed among the cysts of germ cells have unique transcriptional properties. Our current work does not directly address the function(s) of these cells. However, it is intriguing to speculate on their possible roles based on knowledge of the polyploid nurse cells present in the female germ line. Specifically, nurse cells, which undergo 10–12 rounds of endoreplication by the end of oogenesis in *D*. *melanogaster*, produce large amounts of specific RNAs and proteins that are actively transported through cytoplasmic bridges into the developing oocyte^39^. If trophocytes function in transporting needed factors to the germ cells, then their transport system would need to be different from that between the oocyte and its nurse cells because the trophocytes are not physically connected to the germ cells. However, although very speculative, we propose that trophocytes may express “diffusible” factors that help to regulate the overall mitotic/developmental progression of the germ cells of each cyst so that they are coordinated more-or-less together across large regions of the testis. Such an *in trans* means of developmental regulation would be expected to be somewhat imprecise, and is consistent with the variable number of spermatids per bundle within a given testis.

The work presented here adds to a broader understanding of spermatogenesis in hymenopteran insects, especially regarding interesting variation in the cellular dynamics of gametogenesis present within this group. Additionally, these findings provide a number of hypotheses regarding the mechanics and evolution of spermatogenesis in a haploid individual that will be important to test in future studies.

## Materials and Methods

### Wasp lines and husbandry

The majority of our analyses were performed on reproductive tissues taken from male wasps of the AsymC genetic background; however, cytological analysis of a repetitive satellite sequence, PSR4317, was performed on the PSR + genetic background, which contains the supernumerary B chromosome PSR^33^. Wasp lines of the AsympC line were propagated by placing together equal numbers of males and females (10 of each sex) in glass vials containing ~10 *Sarcophaga bullata* blowfly pupae and a few drops of 50% honey in water. PSR crosses were conducted as previously described^40^. Wasps were maintained in an incubator at 25 °C on 12 hr light/dark cycles.

### Procedures for fluorescence confocal microscopy

Wasp pupae were removed from *Sarcophaga bullata* host pupae at the appropriate stage based on wasp pupal color pattern (see Fig. 1 for the three pupal stages used in this study). Testes were dissected in a droplet of 1xPBT (1 × PBS with o.1% Triton-X 100) on a 22 mm plastic Petri plate. Subsequently, the tissues were fixed in a solution of 200 uL 4% paraformaldehyde (diluted in 1 × PBT from a 16% stock solution, Electron Microscopy Sciences) and 600 uL heptane in a 1.5 mL microfuge tube. Fixations were conducted on a platform rocker for 20 min. The heptane and paraformaldehyde mixture was then removed, and tissues were washed 3 times with a full aliquot of 1 × PBT for 5 min each wash while rocking.

Primary antibodies and their dilutions in 1 × PBT were as follows: rabbit anti-H3S10p (Santa Cruz Biotech) at 1:200; mouse anti-acetylated alpha-Tubulin (Santa Cruz Biotech) at 1:100; mouse anti-RNA Polymerase II phosphorylated at Serine residue 2 (Abcam) at 1:500. Secondary antibodies and their dilutions in the same buffer were: anti-rabbit Alexa488 and anti-mouse Cy5 (both at 1:300; Invitrogen, ThermoFisher).

Fixed testes were stained with primary antibodies overnight on a platform rocker at 4 °C, washed three times at 15 minutes each with 1 × PBT. Secondary antibodies were then added to testes and incubated at room temperature on a platform rocker and in the dark (covered with aluminum foil) for 1 hour. The tissues were then washed three additional times with 1 × PBT (still covered) before mounting on a slide with Vectashield mounting medium with DAPI (Vector Laboratories).

DNA fluorescence *in situ* hybridization (FISH) was performed as previously described^33^ using fluorescence labeled DNA oligonucleotide probes synthesized by IDT. PSR22 RNA was detected as in^33^, using a DIG-labeled LNA probe (5′-AATATCCAATCATAAGTCGAGACTTT-3′) generated by Exiqon. FISH was performed using the procedures and reagents provided by the manufacturer in the miRCURY kit. 28S ribosomal RNA and vasa RNA were detected using antisense DIG-labeled RNA probes synthesized *in vitro* from PCR-generated templates via conventional techniques^41^.

Fluorescence confocal microscopy was conducted with a Leica TCS SPE confocal microscope. Images were collected either as a single image, or as Z-series and subsequently merged to capture for inclusion of details in different focal planes. All images were exported in JPEG format and then processed in Adobe Photoshop CS5 v. 12.

### Procedures for transmission electron microscopy

The fixative used was Karnovsky’s Cacodylate Buffered Glutaraldehyde-Formaldehyde (1965), which consists of 3% glutaraldehyde and 3% formaldehyde in 0.1 M cacodylate buffer. All the testes were dissected while the pupa were immersed with fixative at room temperature. The testes within each group were placed into a 1.5 mL microfuge tube filled with fixative, left for 1 hour at room temperature, and then placed at 4 °C overnight. The next day, the samples were warmed to room temperature, the excess fixative removed, and a small amount of low melt agarose (Sigma, Type VII, A9045) added and mixed to disperse the testes. After letting the agarose cool, the agarose blocks were cut out, removed from the microfuge tubes, and placed into fresh fixative. While in new fixative, the agarose blocks were cut in half longitudinally, and the 2 pieces were placed into vials with new fixative. The vials were then kept at 4 °C overnight. The next day, the samples were warmed to room temperature, washed with 0.1 M cacodylate buffer and then post-fixed in 1% osmium tetroxide in 0.1 M cacodylate buffer for one hour. After washing in 0.1 M cacodylate buffer, the samples were dehydrated to 50% ethanol and then en bloc stained in 5% uranyl acetate and 50% ethanol for 60 minutes. The samples were then dehydrated in a graded ethanol series to 100% and then infiltrated with Epon 812 with propylene oxide as the transition fluid. After polymerization at 60 °C for 48 hours, the blocks were ultrathin sectioned, and the sections were stained in uranyl acetate and lead citrate. The sections were viewed in a Hitachi H7000 TEM at 75 kV. Images were captured using an AMT (Advanced Microscopy Techniques, Woburn, MA, USA) 2k × 2k CCD camera. All chemicals were supplied by EMS (Electron Microscopy Sciences, Hatfield, PA USA) unless otherwise specified.

## Supplementary information

Supplementary Information

## Data Availability

The data analyzed in this study are included in this published article.
