# Supplementary material for: Spermatogenesis in haploid males of the jewel wasp Nasonia vitripennis
Source: Sci Rep. 2019 Aug 21;9:12194. doi: 10.1038/s41598-019-48332-9 (PMC6704150; doi:10.1038/s41598-019-48332-9)
Supplement: Supplementary file 1 — Supplementary Information [file 41598_2019_48332_MOESM1_ESM.pdf]

Supplementary Material for:

Title

Spermatogenesis in haploid males of the jewel wasp Nasonia vitripennis

Short Title

Spermatogenesis in the jewel wasp

Authors:

Patrick M. Ferree[1]*, John C. Aldrich[1], Xueyuan A. Jing[1], Christopher T. Norwood[1], Mary R. Van Schaick[1], Manjinder S. Cheema[2], Juan Ausio[2], Brent E. Gowen[3]

Table S1: Expression of Meiosis Genes in *N. vitripennis* Tissues

Meiosis Genes	Accession #	FPKM testis	FPKM ovary	FPKM male soma	FPKM female soma
CORT	NV30291	0.931638	9.4385	0.0854921	0.0341984
DMC1	NV13751	3.78207	1.60682	1.98806	2.41088
HOP2	NV16135	9.66752	60.4494	1.9537	1.79834
MND1	NV13840	9.76098	4.22672	0.91006	2.0806
MSH4	NV16605	3.15871	5.44347	0.201794	0.170351
MSH5	NV22185	0	0	0	0
REC8	NV21644	2.10866	0.115255	0.128652	0.0512485
SPO11	NV18475	1.01396	0.336619	0.174372	0.394835

FPKM stands for Fragments Per Kilobase of transcript per Million reads. These values were obtained from reference 37. Values over 1 FPKM in the germ line tissues are shown in red.

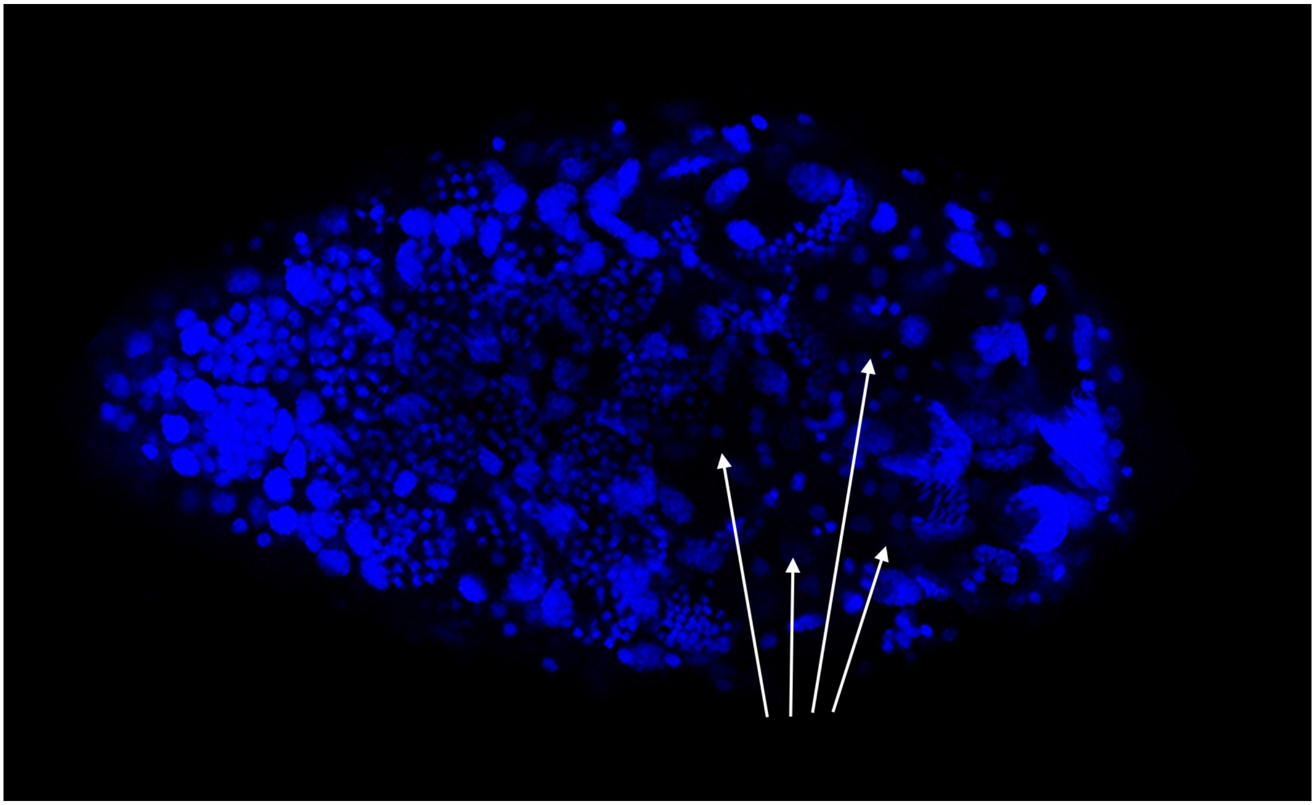

Figure S1. **Negative space toward the posterior region of the testis** (white arrows). Figure 3 shows that these areas of negative space are filled with sperm tails. DNA is blue.
